# Comparison of Laparoscopic and Open Surgery for Women With Early-Stage Epithelial Ovarian Cancer

**DOI:** 10.3389/fonc.2022.879889

**Published:** 2022-04-29

**Authors:** Xuting Ran, Xinlin He, Zhengyu Li

**Affiliations:** ^1^ Department of Gynecology and Obstetrics, West China Second University Hospital, Sichuan University, Chengdu, China; ^2^ Key Laboratory of Birth Defects and Related Diseases of Women and Children (Sichuan University), Ministry of Education, Chengdu, China

**Keywords:** laparoscopy, laparotomy, early-stage ovarian cancer, staging, survival

## Abstract

**Objective:**

This study evaluated the oncologic outcomes of laparoscopy and laparotomy in the management of early-stage ovarian cancer patients.

**Methods:**

We conducted an observational study of women diagnosed with International Federation of Gynecology and Obstetrics (FIGO) 2014 stage I ovarian cancer who underwent surgery at the West China Second University Hospital from 2012 to 2020. Patients who received adjuvant chemotherapy before surgery, those with non-epithelial histopathological types, or those with insufficient data were excluded. Using propensity score matching, data from consecutive laparoscopic patients treated by laparoscopy were matched 1:2 with a cohort of patients undergoing open surgery. The operative and survival outcomes among the matched cohorts were examined using the Kaplan–Meier method.

**Results:**

Among 200 eligible patients, 74 patients undergoing laparoscopy were compared with a cohort of 126 patients undergoing open surgery. Baseline characteristics were similar between groups after matching. Patients who had laparoscopy had a shorter operative time (P = 0.001), a shorter hospital stay (P <0.001), and lower blood loss (P = 0.001) than patients who had open surgery. The median (range) follow-up period was 43.0 (38.8–47.2) and 45.0 (36.0–54.0) months for cases and controls, respectively (P <0.001). There are no significant differences in progression-free survival (P = 0.430, log-rank test) and overall survival (P = 0.067, log-rank test) between the two groups.

**Conclusions:**

There is no difference in prognosis between laparoscopic and open surgery in women with stage I epithelial ovarian cancer. Laparoscopic treatment of early-stage ovarian cancer is safe and feasible for stage I epithelial ovarian cancer patients.

## Introduction

Ovarian cancer (OC) is the second leading cause of gynecologic cancer death in women worldwide, accounting for 4.7% of all cancer deaths in 2020 (1). Although nearly 80% of cases of epithelial ovarian cancer (EOC) are diagnosed as advanced-stage, patients with surgically confirmed stage I ovarian cancer have an optimal prognosis, with an approximately 90% 5-year survival (2). Early-stage EOC patients are treated with comprehensive surgical staging *via* laparotomy, which consists of hysterectomy (non-fertility-sparing cases), bilateral salpingo-oophorectomy, pelvic and para-aortic lymphadenectomy, omentectomy, appendectomy (when indicated depending on histology), peritoneal washings, and peritoneal biopsies (3, 4).

Recently, laparoscopy surgery has grown rapidly in popularity for managing endometrial cancer and cervical cancer and appears to be an attractive option in the surgical management of early-stage EOC (5–7). However, due to a scarcity of high-quality studies, the efficiency and safety of the laparoscopic approach in early EOC remain unknown. Most published studies are small-sized, many of which do not control for possible confounding, lack long-term follow-up and detailed information on surgical and survival outcomes (8–11). More evidence is needed for an evaluation of the long-term effects of laparoscopic staging of early-stage ovarian cancer. This study compared the outcomes of laparoscopic with those of laparotomic treatment of obvious stage I ovarian cancer.

## Materials and Methods

This is a retrospective cohort study recruiting early EOC patients undergoing surgery following the Declaration of Helsinki and approved by the ethics committee of West China Second University Hospital, Sichuan University. We retrieved electronic medical records of all women diagnosed with early EOC who underwent surgery at the West China Second University Hospital, Sichuan University, between 2012 and 2020. Women were eligible for the analysis if they met the following criteria: (1) histologically confirmed ovarian cancer with the four major histology types (serous, mucinous, endometrioid, and clear cell); (2) tumor at FIGO 2014 stage I; (3) undergone adnexectomy as primary surgical treatment; and (4) follow-up information. Stage II–IV, nonsurgical management, neoadjuvant therapy before surgical treatment, insufficient data, borderline ovarian tumors, and patients with synchronous cancer are all criteria for exclusion. The World Health Organization (WHO) taxonomy was used to classify histologic subtypes.

Patient demographics, tumor characteristics, and surgical and postoperative chemotherapy information were abstracted from the medical records. Patient demographics included age, years at diagnosis, comorbidities, BMI, menopause (yes or no), family history of cancer, and history of abdominal surgery. Tumor characteristics include tumor size, histology type, pathologic grade, capsule rupture (yes or no), intraoperative rupture (yes or no), and stage of the disease. Year of surgery, surgical type, operation time, estimated blood loss, length of hospital stay, intraoperative complications, and postoperative chemotherapy (yes or no), were all included in the surgical and chemotherapy information. Oncologic outcome data, namely, survival status, recurrence time and sites, and cause of death, were collected by telephone interview. Follow-up time was up to December 2020.

The exposure of interest was the surgical type. We enrolled subjects whose staging procedure was completed mainly with an open surgical approach in the laparotomy group, irrespective of whether or the procedure was initiated laparoscopically. Furthermore, all these staging surgeries were performed by 15 surgeons with experience in practice between 2012 and 2020 in our institution, and all the surgeons had an equal breakdown for laparoscopic and laparotomic approaches.

Intraoperative cyst rupture was defined as an intentional or unintentional event that resulted in a spill into the peritoneal cavity. If the procedure used a collection bag, the cyst was not considered ruptured. Intraoperative complications are defined as adverse events during surgery, mainly including blood transfusions or conversion to laparotomy. Postoperative complications are defined as adverse events occurring within 30 days of surgery because of the procedure. The length of hospital stay was calculated from the first postoperative day.

The primary outcome was overall survival, defined as months from cancer diagnosis to death, or the date of the telephone interview. Secondary outcomes included the cumulative survival at three and four years after diagnosis. We also compared the length of hospital stay, estimated blood loss, intraoperative and postoperative complication rates, and cyst rupture rates.

To reduce bias in the survival estimates of the two surgical approaches, we used the propensity score matching (PSM) method in a 1:2 ratio to match patients who have undergone laparoscopy to those treated with open surgery. Covariates, including age at diagnosis, tumor pathological type, tumor grade, and receipt of any adjuvant chemotherapy, were categorized as yes, no, or unknown. We grouped tumors into sizes of 1.0–4.9, 5.0–9.9, and 10.0–14.9 cm or larger, and 1 cm increments for the calculation of propensity scores. The extent of comorbidity was categorized as zero, one, or more than one comorbidity, using the Deyo adaptation of the Charlson comorbidity index. Furthermore, since there has been an increase in MIS use over the years, the years of surgery were grouped as 2012.1–2014.12, 2015.1–2017.12, and 2018.1–2020.1.

Given the significantly better prognosis of mucinous ovarian cancers and the fact that they are often larger in size. We stratified the propensity-matched cohort by histology of mucinous type and repeated the primary survival analysis in each group to evaluate whether the effect of planned laparoscopy was sensitive to mucinous ovarian cancers.

Statistical analysis was performed using SPSS software version 23 (IBM Company, Armonk, NY, USA). χ^2^ test was used for categorical variables. Patients undergoing laparoscopic approach were matched 1:2 to the laparotomy group, using a caliper width of 0.1 standard deviations (SDs) of the logit of the propensity score. A Kaplan–Meier survival analysis and a log-rank test were used to describe the overall survival (OS) and progression-free survival (PFS) differences between groups. A statistical difference was considered significant when the *P*-value was <0.05.

## Results

**Figure 1:**
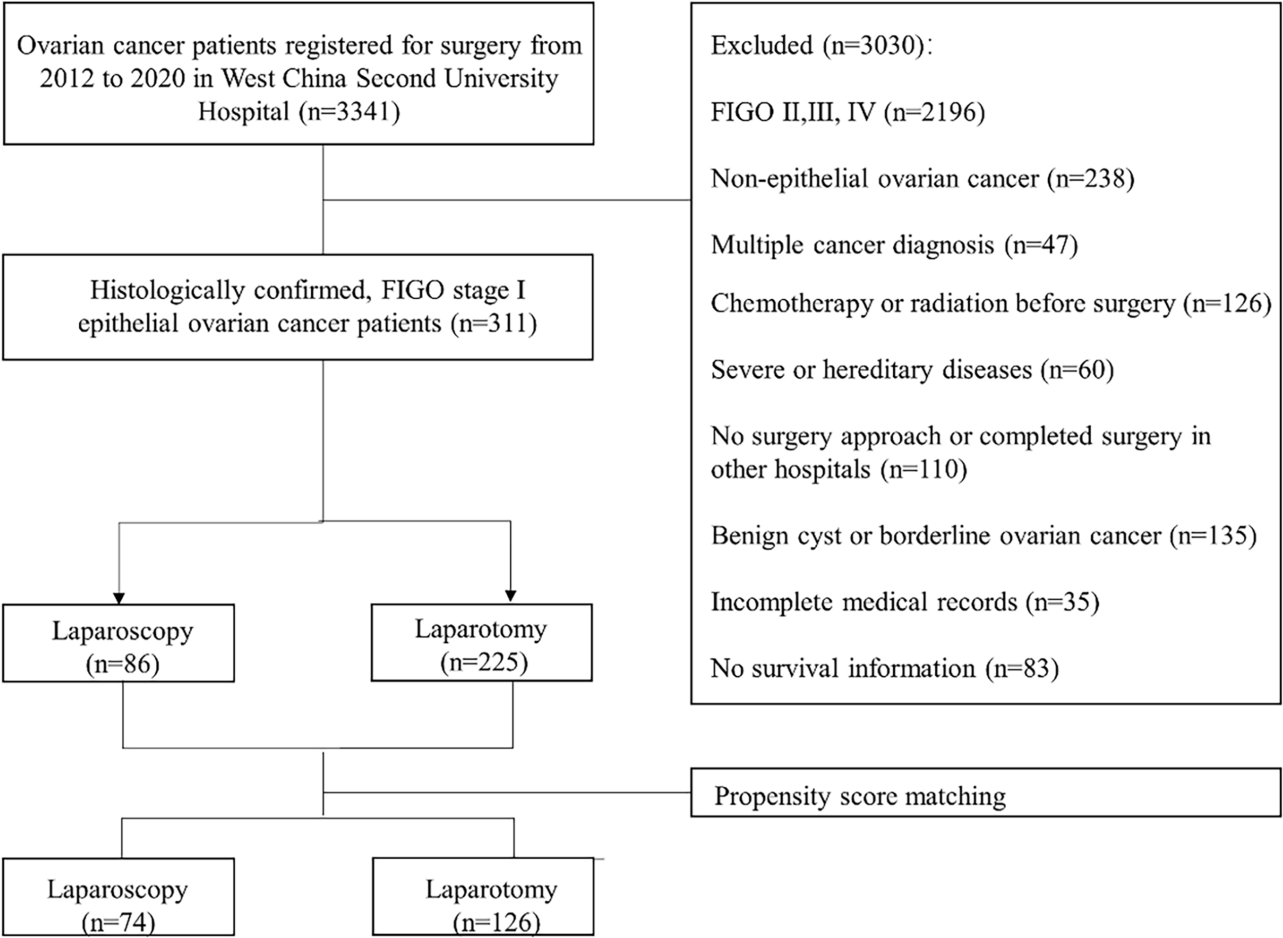
Flow chart of patients’ selection.

A total of 311 patients met the eligibility criteria, among whom 225 patients underwent laparotomy and another 86 underwent laparoscopy for treatment. After 1:2 propensity score matching, 74 patients who underwent laparoscopy were successfully matched with 126 patients who underwent laparotomy. The study cohort flow is shown in **Figure 1**.

Baseline characteristics of the study groups are presented in **Table 1**. Before PSM, tumor sizes were smaller in the laparoscopy group before PSM compared to the laparotomy group. After the application of PSM, baseline characteristics were similar between groups, including covariates of age, comorbidity, histological type, grade, tumor size, stage, and adjuvant chemotherapy (Paclitaxel plus platinum-based chemotherapy). Six patients followed bevacizumab for maintenance. Likewise, there were no significant differences among other demographic characteristics containing menopause, BMI, ascites, lymph node enlargement, abdominal surgery history, and family history of cancer, suggesting that the two groups were well balanced after propensity-matching. Moreover, we found that two patients had a BRCA mutation in those who underwent genetic testing. The balance of covariates was confirmed by absolute standardized differences, which was less than 10% after PSM (**Figure S1**).

**Table 1:** Characteristics of patients undergoing surgery of presumed stage I epithelial ovarian cancer surgical approach before and after propensity score matching.

Characteristic		Before PSM			After PSM		
	Laparoscopy (n = 86)	Laparotomy (n = 225)	*P*	Laparoscopy (n = 74)	Laparotomy (n = 126)	*P*
Median age, yrs (interquartile rage)	49 (44–56)	48 (42–53)	0.165^a^	48.5 (43–54)	48 (44–54)	0.815^a^
Charlson Comorbidity Index			0.388^b^			0.880^b^
0	52 (60.5)	106 (47.1)		41 (55.4)	68 (54.0)	
1	19 (22.1)	61 (27.1)		19 (25.7)	36 (28.6)	
2 or more	15 (17.4)	58 (25.8)		14 (18.9)	22 (17.4)	
Histological type			0.026^b^			0.407^b^
Clear cell	41 (47.7)	89 (39.6)		37 (50.0)	54 (42.9)	
Endometrioid	10 (11.6)	32 (14.2)		10 (13.5)	18 (14.3)	
Serous	20 (23.3)	29 (12.9)		16 (21.6)	21 (16.7)	
Mucinous	9 (10.5)	49 (21.8)		7 (9.5)	23 (18.3)	
Other	6 (7.0)	26 (11.6)		4 (5.4)	10 (7.9)	
Histological grade			0.011^b^			0.236^b^
1	13 (15.1)	66 (29.3)		13 (17.6)	31 (24.6)	
2	2 (2.3)	16 (7.1)		2 (2.7)	10 (7.9)	
3	60 (69.8)	125 (55.6)		53 (71.6)	75 (59.5)	
Unknown	11(12.8)	18(7.6)		6 (8.1)	10 (7.9)	
Tumor size (cm)			<0.001^b^			0.071^b^
1.0–4.9	6 (7.0)	5 (2.2)		3 (4.1)	5 (4.0)	
5.0–9.9	27 (31.4)	41 (18.2)		21 (28.4)	31 (24.6)	
10.0–14.9	27 (31.4)	52 (23.1)		24 (32.4)	37 (29.4)	
15 or larger	10 (11.6)	88 (39.1)		10 (13.5)	38 (30.2)	
Unknown	16 (18.6)	39 (17.3)		16 (21.6)	15 (11.9)	
Adjuvant chemotherapy			0.912^b^			0.746^b^
Yes	66 (76.7)	174 (77.3)		59 (79.7)	98 (77.8)	
Unknown	20 (23.3)	51 (22.7)		15 (20.3)	28 (22.2)	
Menopause	38 (44.2)	96 (42.7)	0.898^b^	36 (48.6)	51 (40.5)	0.260^b^
BMI (kg/m^2^)	22.79 ± 3.1	23.05 ± 3.4	0.554^b^	22.86 ± 3.2	23.03 ± 4.3	0.762^b^
FIGO stage			0.197^b^			0.744^b^
IA	42 (48.8)	135 (60.0)		41 (55.4)	65 (51.6)	
IB	5 (5.8)	12 (5.3)		4 (5.4)	5 (4.0)	
IC	39 (45.3)	78 (34.7)		29 (39.2)	56 (44.4)	
Ascites^c^	20 (23.3)	74 (32.9)	0.224^b^	18 (31.0)	45 (44.1)	0.130^b^
Lymph nodes enlargement^c^	4 (4.7)	26 (11.6)	0.119^b^	8 (10.8)	14 (11.1)	0.143^b^
Abdominal surgery history	35 (40.7)	97 (43.1)	0.703^b^	28 (37.8)	62 (49.2)	0.141^b^
Family history of cancer	16 (12.0)	27 (18.6)	0.144^b^	11 (14.9)	15 (11.9)	0.664^b^

a Mann–Whitney non-parametric test.

b χ2 test.

c 46 missing.

The operative outcomes are shown in **Table 2**. The laparoscopy group had a shorter length of hospital stay (6 days vs. 7 days; *P <*0.001), shorter operation time (269.12 [ ± 78.98] vs. 313.08 [ ± 96.77]; *P <*0.001), and lower blood loss (254.46 [ ± 309.49] vs. 424.26 [ ± 293.46]; *P <*0.001). However, the cyst rapture rate was higher in the laparoscopy group than in the laparotomy group. There were no significant differences in the number of hysterectomy cases or in the pelvic and para-aortic lymph nodes removed. No intraoperative complications occurred in the laparoscopy group, and one patient experienced hemorrhage during open surgery. One patient in the laparoscopy group and 2 patients in the laparotomy group developed a fever on the second day after surgery. The other 2 patients were diagnosed with thrombosis by ultrasound postoperatively.

**Table 2:** Operative outcomes by surgery approach in propensity score-matched cohort.

Surgical outcome	Laparoscopy (n = 74)	Laparotomy (n = 126)	*P*
Length of hospital stay (days), median (IQR)	6 (5–7.75)	7 (6–9)	<0.001^a^
Operation time (mins)	269.12 (78.98)	313.08 (96.77)	0.001^b^
Estimated blood loss (ml)	254.46 (309.49)	424.26 (293.46)	0.001^b^
Blood transfusion (n)	1 (1.4)	9 (7.1)	<0.001^c^
Hysterectomy (n)	67 (90.5)	120 (95.2)	0.238
Pelvic lymphadenectomy (n)	67 (90.5)	116 (92.1)	0.794^c^
Para-aortic lymphadenectomy (n)	54 (73.0)	85 (67.5)	0.432^c^
Intraoperative complication (n)	0	1 (0.8)	NS
Cyst rupture (n)^d^	19 (29.2)	12 (10.2)	0.002^d^
Postoperative complication (n)^e^	2 (5.3)^g^	3 (4.7)	NS

a Mann–Whitney non-parametric test.

b t-test.

c χ^2^ test.

d 17 missing.

e Postoperative complications includes fever, venous thrombosis, intestinal obstruction and poor wound healing. IQR, Intraquartile range; NS, No significant.

**Figure 2:**
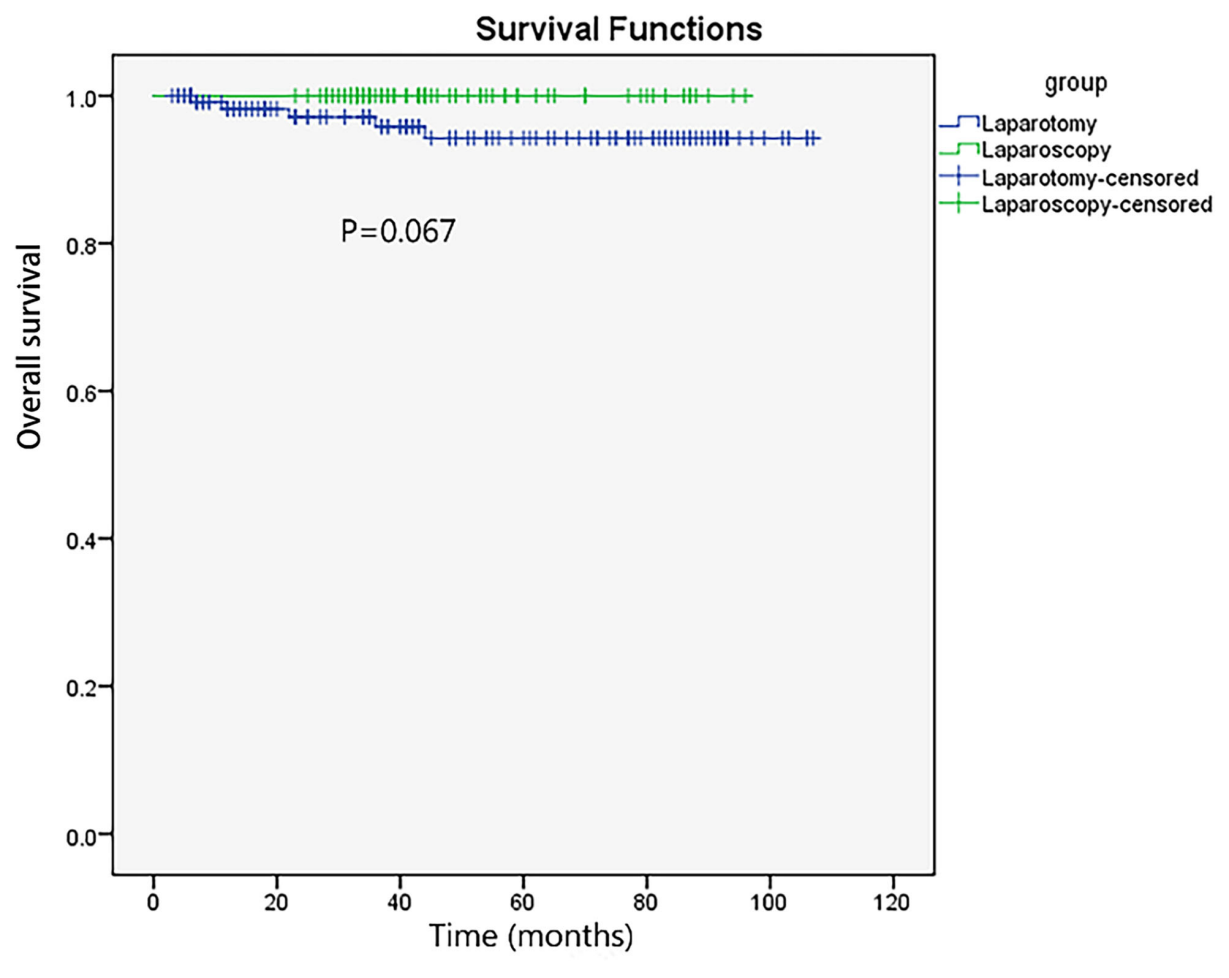
Overall survival of laparoscopy and laparotomy in patients with early EOC.

**Figure 3:**
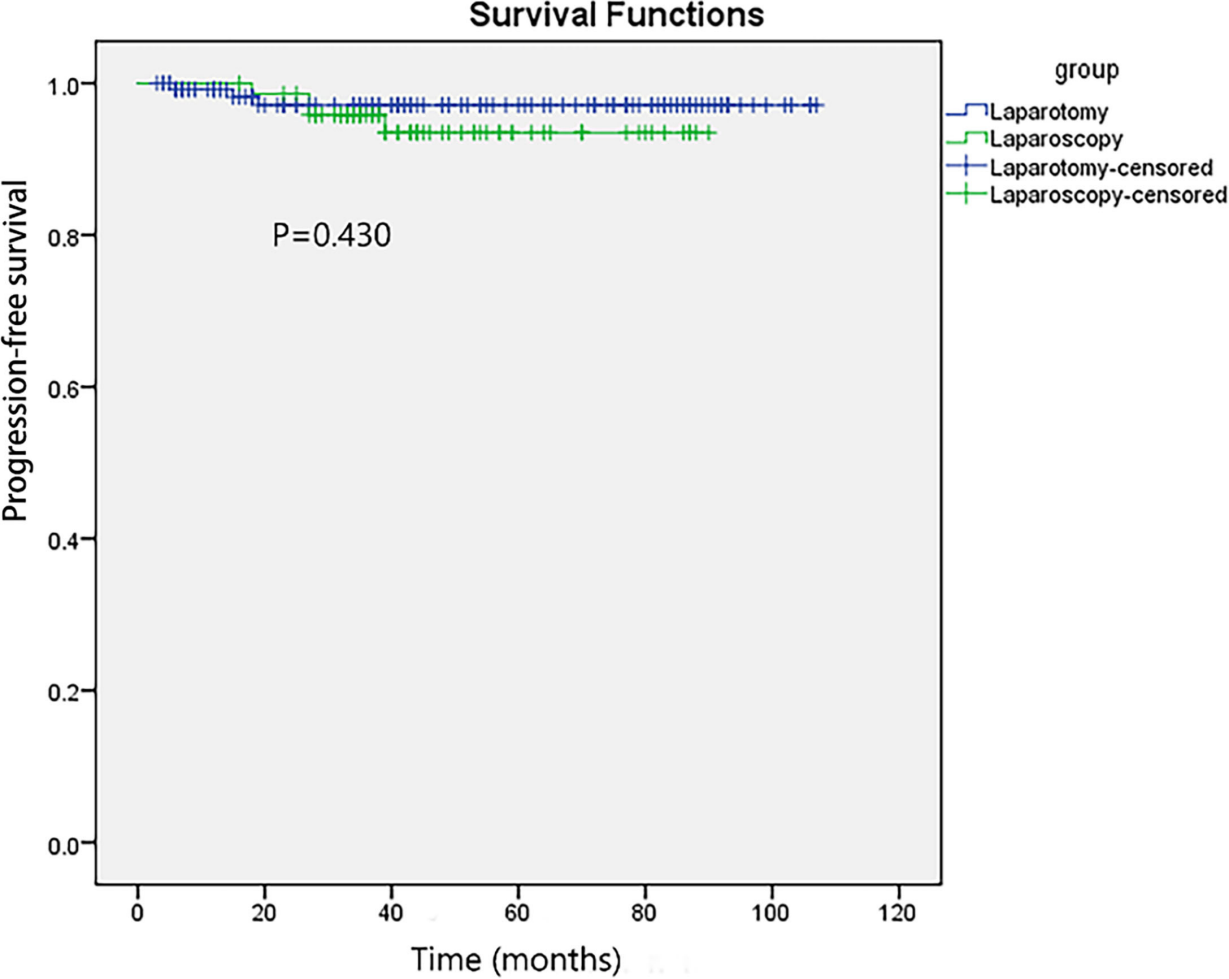
Progression-free survival of laparoscopy and laparotomy in patients with early EOC.

The median follow-up time was 43.0 (38.8–47.2) and 45.0 (36.0–54.0) months in the laparoscopy and laparotomy groups, respectively (*P <*0.001). Overall survival (*P* = 0.067, log-rank test) (**Table 3** and **Figure 2**) and progression-free survival (*P* = 0.430, log-rank test) (**Figure 3**) were similar between the two groups. The 1- and 3-year progression-free survival rates were 98.2% and 95.9%, respectively, in the laparoscopy group, and no deaths occurred in the laparotomy group. Five patients died of tumor metastasis regardless of postoperative chemotherapy. In the laparoscopy group, four patients developed recurrence in the sigmoid colon, abdominal lymph nodes, and peritoneum by PET/CT, and they were also observed to have elevated tumor biomarkers. Three recurrences in the sigmoid colon, mesentery, and abdominal lymph nodes were observed in the laparotomy group.

**Table 3:** Comparison of survival outcomes and stratified surgical, histological types in propensity score-matched cohort.

Survival outcomes	Laparoscopy (n = 74)		Laparotomy (n = 126)		*P*
Median follow up, months (95%)	43.0 (38.8–47.2)		45.0 (36.0–54.0)		0.089^a^
Progression free survival probability, percent (95% CI^a^)					
1 year	98.2 (0.013)	100			0.13^b^
3 years	95.8 (0.021)	100			0.06^b^
Recurrence (n)	4 (5.4)	3 (2.4)			NS
Overall survival	100%	96.0%			NS
Death (n)	0 (0)	5 (4.0)			NS
Stratified surgical types (n)	No. of events	Median follow-up, months (95%)	No. of events	Median follow-up, months (95%)	
One-step surgery/death	55 (74.3)/0 (0)	42.1 (38.6–47.2)	97 (77.0)/5 (4.0)	44.4 (36.2–47.0)	0.106^a^
Two-step surgery/death	19 (25.7)/0 (0)	41.2 (39.5–46.3)	29 (23.0)/0 (0)	45.2 (37.0–51.6)	0.393^a^
Fertility-sparing surgery/death	4 (5.4)/0 (0)	–	3 (2.4)/0 (0)	–	NS
Complete surgical staging surgery/death	70 (94.6)/0 (0)	41.0 (37.2–47.4)	123 (97.6)/5 (4.0)	45.0 (36.3–52.0)	0.075^a^
Stratified histological types (n)					
Without mucinous type/death	60 (81.1)/0 (0)	40.3 (38.7–44.9)	99 (78.6)/5 (4.0)	45.2 (35.5–54.0)	0.061^a^

a Calculated using the Kaplan–Meier method.

b Z-test for independent proportions. CI, Confidence interval; NS, Not significant.

We further analyzed survival outcomes between laparoscopy and laparotomy groups by stratifying surgical types (**Table 3**). Nineteen patients and 29 patients underwent two-step surgery in the laparoscopy and laparotomy groups, respectively, and there was no significant difference in the OS rate between the two groups (*P* = 0.393). Four patients in the laparoscopy group and 3 patients in the laparotomy group underwent fertility-sparing surgery, and the overall survival rate was 100% in both groups. For other patients treated with complete surgical staging surgery, the overall survival rate also had no significant difference (*P* = 0.075). Furthermore, after stratifying the study population by histology of mucinous type, we found no difference in survival between patients who underwent staging by laparoscopy and laparotomy, irrespective of mucinous histology (*P* = 0.061).

## Discussion

In this retrospective study, we compared the survival outcomes of patients with FIGO stage I EOC using different surgical approaches. The results showed no difference in OS and PFS between patients who underwent laparoscopy treatment and those who underwent laparotomy treatment. The laparoscopy approach was associated with shorter operation time, shorter length of hospital stay, lower blood loss, and higher cyst rupture rate compared to the laparotomy approach. By propensity score matching, we adjusted covariates for comorbidities, receipt of adjuvant chemotherapy, pathologic stage, grade, histological types, and tumor size to mimic a randomized study.

Laparoscopic surgery has become common in gynecological practice recently. However, the evidence for the use of laparoscopy use in early-stage ovarian cancer remains insufficient. In 2016, the Cochrane Database of Systematic Reviews found no randomized controlled trials (RCTs) and scarce data in high-quality evidence to compare the effect of laparoscopy use with conventional laparotomy (12), since laparoscopy is a relatively new technique for treating stage I ovarian cancer and there were difficulties in recruiting sufficient patients to perform RCTs. After that, several studies have evaluated the safety and effectiveness of laparoscopy use in OC. Our findings follow previous studies, which indicated that there was no survival difference for women treated with laparoscopy compared with laparotomy (11, 13–18). A meta-analysis including these 6 studies also reported that there were no differences in 4–5 year OS and PFS between these two surgical approaches, and another meta-analysis, which included 10 studies, also reported similar results (19, 20). There are also several concerns about the laparoscopy approach in the staging of the early OC.

cern is the high risk of cyst rupture during the laparoscopy procedure, which may upstage the tumor from stage IA to IC and, theoretically, increase mortality. However, previous studies have found different outcomes for cyst ruptures following laparoscopic and laparotomy surgeries. Many studies have reported that the cyst rupture rate was similar between the laparoscopic and laparotomic approaches and that there was no significant difference in survival outcomes (15, 21–23). In contrast, a recent study by Matsuo et al. involving 2,600 women who underwent laparoscopic and robotic-assisted surgery, reported that laparoscopy was independently associated with cyst rupture, increasing all-cause mortality (24). Besides, a large, multicenter study including 1,545 patients reported that rupture during surgery may be associated with a poor prognosis for women with stage I EOC (25). Similarly, Koji et al. found that intraoperative rupture was associated with a worse effect on survival in 15,163 women with stage IA–IC1 OC (26). In our study, we found that the cyst rupture rate was significantly higher in the laparoscopy group than in that of the laparotomy group, but there was no difference in survival between the two groups. Due to the small scale of our study, larger and prospective studies are needed to examine the prognostic effect on cyst rupture and survival. Efforts are needed to reduce tumor spillage, including the selection of smaller tumor-sized patients, use of a laparoscopic bag, controlled aspiration, and careful separation.

Lymphadenectomy is another concern of the laparoscopic approach. The number of excised lymph nodes has a prognostic value and is related to upstage rates in early-stage OC (27). In this study, lymphadenectomy was similar between the laparoscopy and the laparotomy groups. Previous studies also found no between-group differences in the number of total, para-aortic, and pelvic lymph nodes retrieved (16, 28).

Port-site metastasis is also a great controversy. The incidence of port-site metastasis in laparoscopic use is less than 1% in early-stage ovarian cancer, much lower than in advanced OC (6). In their meta-analysis, which contained 11 studies (29), Park et al. reported only 1 patient who experienced port-site metastasis of laparoscopy use in early-stage OC in their meta-analysis. In this study, we found no port-site metastasis. Ataseven et al. reported that port-site metastasis had no impact on survival. However, port-site metastasis was associated with more postoperative complications and a higher surgical treatment burden. This should be balanced with the expected benefit when laparoscopy is considered for managing EOC (30, 31).

Because some patients did not finish staging surgery immediately after bilateral salpingo-oophorectomy, we further stratified the surgical procedure into one-step and delayed, two-step surgeries, and then compared the survival outcomes between the laparoscopy and the laparotomy groups. We found no significant differences in survival, suggesting immediate or delayed complete staging surgeries are both safe options for stage I EOC. Besides, several studies have evaluated the feasibility and safety of fertility-sparing staging surgery using a laparoscopic approach in early-stage EOC (32–34). Here we also tried stratifying and comparing the efficacy of fertility-sparing staging surgery between laparoscopy and laparotomy. However, we failed to make an effective evaluation due to the small number of patients. Multicenter, larger studies are needed for both considerations of oncological and pregnancy outcomes for laparoscopic surgery in patients seeking fertility preservation.

The strength of our study is the record of relatively more detailed surgical outcomes of the patients. Additionally, adjustment for related biases of demographic information, morbidities, adjuvant chemotherapy, and tumor information of the patients improved the value of our results. Furthermore, all surgeries are performed by skilled surgeons in gynecologic oncological surgery, which ensures consistency of procedure. Nonetheless, there are several limitations that must be considered in interpreting the findings. A limitation of our study was its retrospective design, which possibly introduced some degree of bias. Although we used PSM to mimic a randomized study, it does not circumvent the issue of decisions of the surgeons regarding surgical approach selection. A prospective randomized trial is needed to clarify the role of laparoscopic staging for ovarian cancer.

## Conclusions

There is no difference in survival outcomes between laparoscopy and laparotomy in the management of stage I EOC. The benefits of laparoscopy are that it may shorten the hospital stay and cause less blood loss. In this way, laparoscopic staging of early EOC is a feasible and safe approach for selected patients. Larger, prospective studies are still needed to confirm these findings.

## Data Availability Statement

The original contributions presented in the study are included in the article/**Supplementary Material**. Further inquiries can be directed to the corresponding author.

## Ethics Statement

The studies involving human participants were reviewed and approved by the Ethics Committee of West China Second University Hospital, Sichuan University. The patients/participants provided their written informed consent to participate in this study.

## Author Contributions

XR: methodology, formal analysis, investigation, writing—original draft preparation. XH: methodology, formal analysis, investigation. ZL: conceptualization, formal analysis, investigation, resources, data curation, writing—review and editing, supervision, project administration, funding acquisition. All authors listed have made a substantial, direct, and intellectual contribution to the work and approved it for publication.

## Funding

This study was supported by the Medical Science and Technology Project of Sichuan Provincial Health Commission (grant number 21PJ050).

## Conflict of Interest

The authors declare that the research was conducted in the absence of any commercial or financial relationships that could be construed as a potential conflict of interest.

## Publisher’s Note

All claims expressed in this article are solely those of the authors and do not necessarily represent those of their affiliated organizations, or those of the publisher, the editors and the reviewers. Any product that may be evaluated in this article, or claim that may be made by its manufacturer, is not guaranteed or endorsed by the publisher.
