# Supplementary material for: Comparison of Laparoscopic and Open Surgery for Women With Early-Stage Epithelial Ovarian Cancer
Source: Front Oncol. 2022 Apr 29;12:879889. doi: 10.3389/fonc.2022.879889 (PMC9098929; doi:10.3389/fonc.2022.879889)
Supplement: Supplementary file 1 [file DataSheet_1.docx]

Supplementary Material

## Supplementary Figures


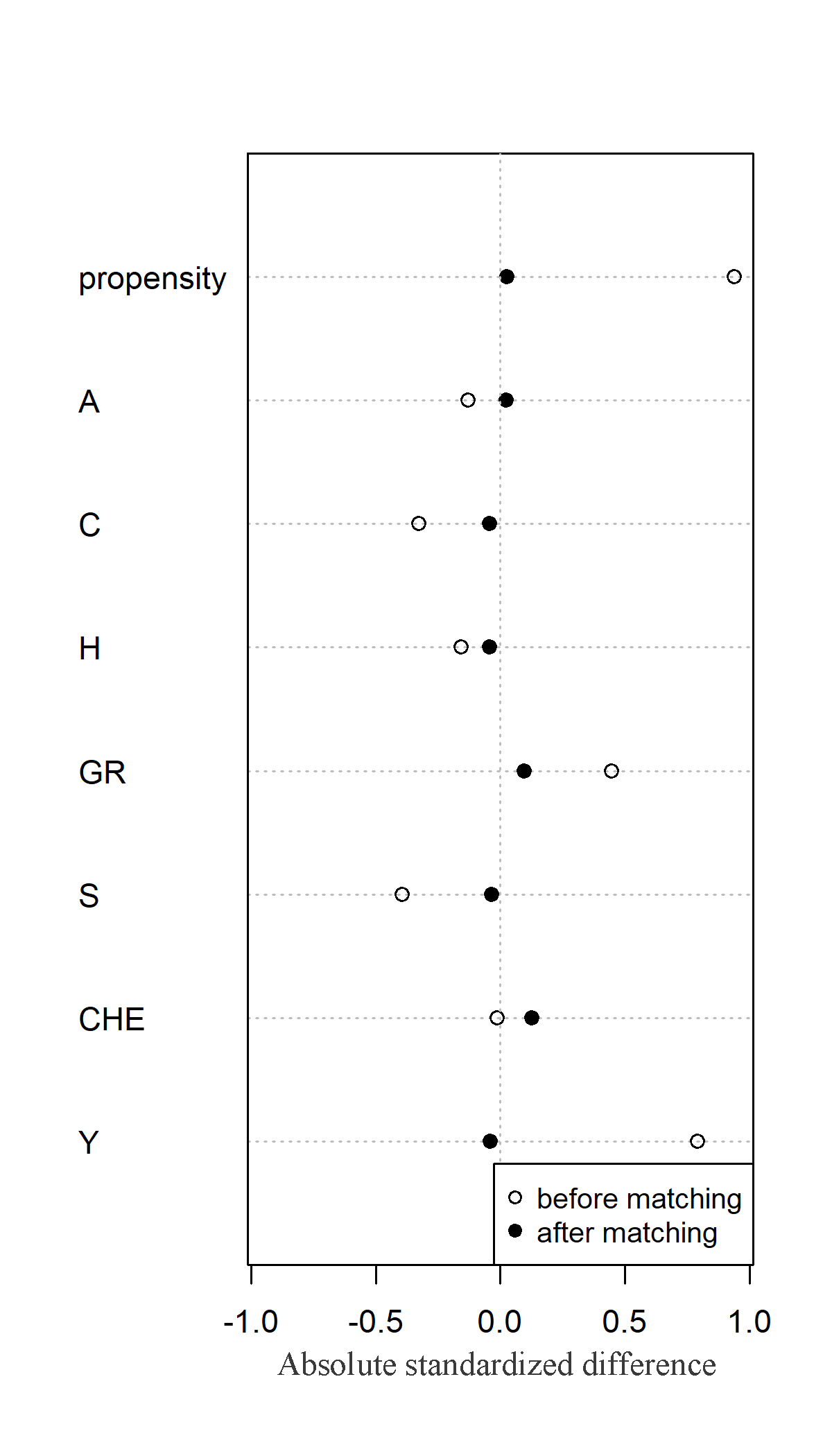


**Supplementary Figure 1.** Absolute standardized differences in variables included in propensity score before (solid diamonds) and after (hollow circles) propensity score matching. Groups are considered well balanced on a covariate when the absolute standardized difference is less than 0.1. Abbreviations: A: age; C: comorbidity, H: histological type, GR: grade; S:tumor size, CHE: adjuvant chemotherapy, Y: year of surgery
